# Precordial pain caused by a hemocholecyst due to gallbladder cancer: A case report

**DOI:** 10.1016/j.ijscr.2022.107851

**Published:** 2022-12-23

**Authors:** Goshi Fujimoto

**Affiliations:** Department of Gastroenterological Surgery, Koga Community Hospital, Yaizu, Shizuoka, Japan

**Keywords:** Chest pain, Gallbladder cancer, Haemobilia, Haemocholecyst, Precordial pain

## Abstract

**Introduction and importance:**

A hemocholecyst refers to hemorrhage originating from and confined to the gallbladder. Intraluminal hemorrhage of the gallbladder is a rare symptom of gallbladder cancer (GBC), which can cause hemorrhagic cholecystitis. The symptoms of hemorrhagic cholecystitis are similar to those of classic acute cholecystitis where precordial pain is atypical. Here, we report a case of a precordial pain-inducing hemocholecyst due to GBC.

**Case presentation:**

An 86-year-old woman was admitted to the emergency department due to persistent, sudden-onset precordial pain. Electrocardiogram (ECG) findings and cardiac enzyme levels were normal; however, severe anemia (hemoglobin 6.4 g/dL) was noted. Computed tomography (CT) showed a tense gallbladder with a heterogeneous, high-density area. Contrast-enhanced CT did not reveal contrast extravasation or obvious mass lesions. Considering the risk of hemorrhagic cholecystitis, we performed laparoscopic cholecystectomy. Operative findings were normal, however, the gallbladder lumen was filled with blood clots, while the gallbladder body had a papillary, infiltrating-type lesion.

**Clinical discussion:**

Histopathological examination confirmed the diagnosis of moderately differentiated gallbladder adenocarcinoma. The precordial pain disappeared postoperatively. Due to the patient's age and general condition, no additional gallbladder bed resection or S4/5 hepatic bisegmentectomy and lymphadenectomy were performed.

**Conclusion:**

A hemocholecyst can cause precordial pain; therefore, abdominal imaging may be useful for diagnosing patients with nonspecific precordial pain. In addition, GBC should be considered as a potential cause of hemocholecysts. Early diagnosis and urgent cholecystectomy should be performed to prevent gallbladder perforation in patients with hemocholecysts.

## Highlights

• Intraluminal hemorrhage of the gallbladder is a rare symptom of gallbladder cancer.
• A hemocholecyst can cause chest pain.
• Chest pain (rare) in cholecystitis may be due to the cardiobiliary vagal reflex.
• Early diagnosis and urgent cholecystectomy can prevent gallbladder perforation.

## 1 Introduction

Hemobilia is a condition in which trauma or a disease causes hemorrhage into the biliary tract, thereby leading to the flow of blood and bile into the duodenum. Hemorrhage originating from and confined to the gallbladder is referred to as a hemocholecyst. The most common cause of hemobilia due to a gallbladder disease is cholecystolithiasis, the presence of gallstones in the gallbladder neck, which causes gallbladder artery erosion [1]. Intraluminal gallbladder hemorrhage is a rare symptom of gallbladder cancer (GBC) occurring in only 1 % of patients [2]. Hemorrhagic cholecystitis develops when the gallbladder bulges and blood clots clog the gallbladder duct [3], [4]. The symptoms of hemorrhagic cholecystitis are similar to those of classic acute cholecystitis, where precordial pain is atypical [5]. Precordial pain has not been reported previously as a symptom of hemocholecysts. Rarely, patients with acute cholecystitis associated with chest pain undergo invasive cardiac examinations which may delay the diagnosis of cholecystitis. Here, we report a case of a precordial pain-inducing hemocholecyst due to GBC. This case has been reported in line with the SCARE 2020 criteria [6].

## 2 Case presentation

**Fig. 1:**
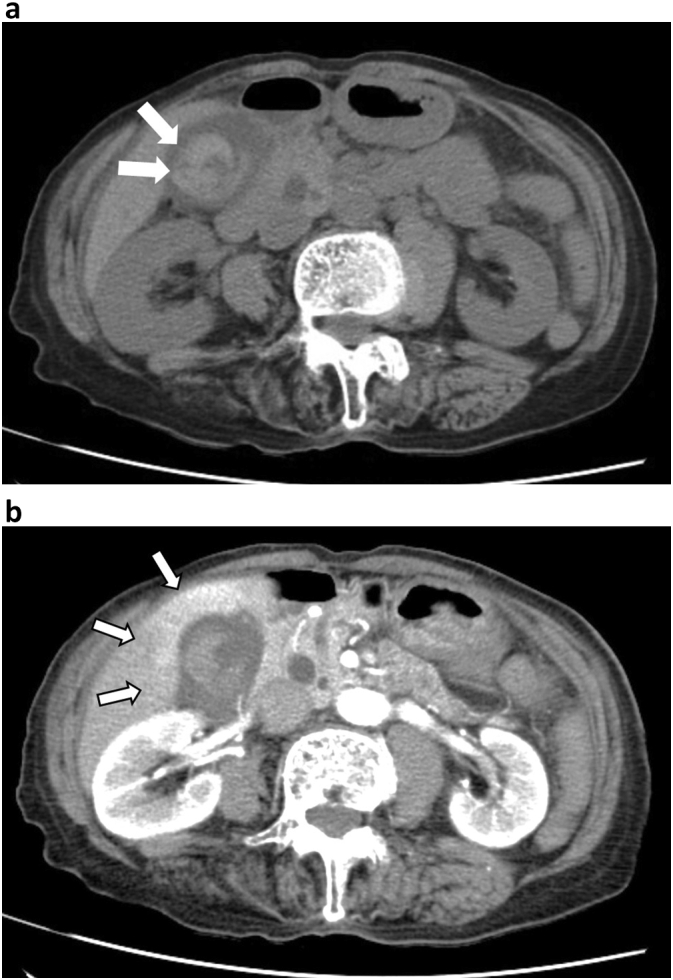
a. Findings on computed tomography. Computed tomography showed a tense gallbladder with a heterogeneous, high-density area (arrows). b. Findings on contrast-enhanced computed tomography in the arterial phase. Contrast-enhanced computed tomography showed increased pericholecystic attenuation in the arterial phase (arrows), which suggests acute cholecystitis.

**Fig. 2:**
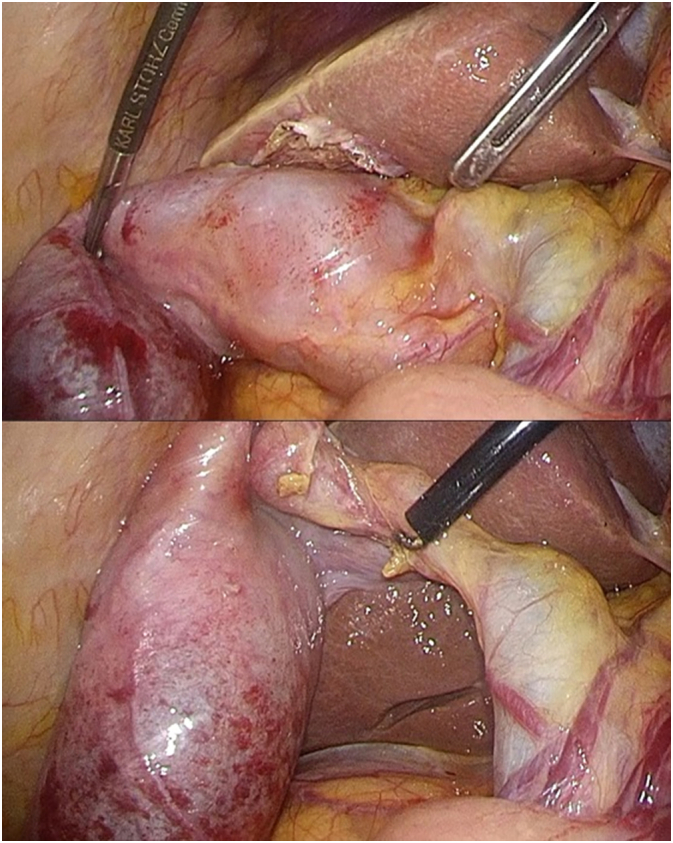
Operative findings. Operative findings did not reveal any evidence of malignancy in the gallbladder serosa.

An 86-year-old woman (body mass index of 20.5 kg/m^2^) with a history of depression was admitted to the emergency room due to sudden-onset, non-radiating precordial pain lasting 9 h. Dyspnea, nausea, and back pain were not reported. She was taking escitalopram 5 mg as needed and had no family history of GBC. On physical examination, her abdomen was soft, flat, and scar-free. Electrocardiogram (ECG) did not show ST segment changes. On blood examination, her cardiac enzyme, white blood cell, and C-reactive protein levels were not elevated; however, her hemoglobin level was low (6.4 g/dL). Computed tomography (CT) revealed a tense gallbladder with a heterogeneous, high-density area (Fig. 1a) with no evidence of pleurisy or aortic dissection. Contrast-enhanced CT showed no obvious contrast extravasation in the gallbladder or obvious mass lesions. Increased pericholecystic attenuation on arterial-phase CT was confirmed, which indicated the possibility of acute cholecystitis (Fig. 1b). Due to the risk of acute cholecystitis and gallbladder perforation, we performed laparoscopic cholecystectomy without lymphadenectomy. Operative findings did not reveal any evidence of malignancy in the gallbladder serosa. Congestive findings were noted from the body to the fundus of the gallbladder (Fig. 2). The total operating time was 47 min, while the intraoperative blood loss was 5 mL. Two units of red blood cells were transfused intraoperatively. The patient had no postoperative complications.

**Fig. 3:**
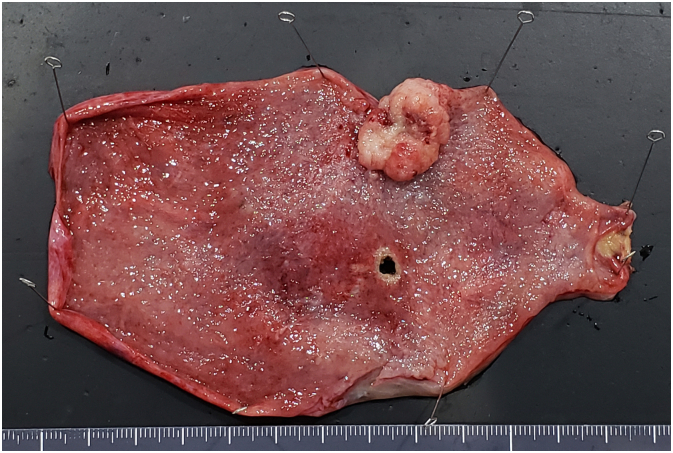
Macroscopic findings of the specimen. A papillary, infiltrating-type lesion measuring 25 × 17 mm was observed in the body of the gallbladder.

Examination of the resected specimen revealed a blood clot-filled gallbladder lumen and a gallbladder body with a papillary, infiltrating-type lesion measuring 25 × 17 mm (Fig. 3). Histopathological examination revealed moderately differentiated adenocarcinoma of the gallbladder invading perimuscular connective tissue on the peritoneal side with no extension to the serosa (T2a according to the 8th edition of the UICC TNM classification).

The precordial pain disappeared postoperatively. Although the tumor depth was T2a, considering her age and general condition, the patient and her family decided to forgo additional gallbladder bed resection or S4/5 hepatic bisegmentectomy and lymphadenectomy.

## 3 Clinical discussion

Hemobilia due to a gallbladder disease is rare and can result from cholelithiasis, hemorrhagic cholecystitis, GBC, proliferative cholecystitis, and aberrant pancreatic tissue in the gallbladder wall [1], [4]. The clinical presentation of hemobilia is known as Quincke's triad, which includes jaundice, gastrointestinal bleeding, and abdominal pain. Since these findings are present in only 22–35 % of cases, hemobilia is difficult to diagnose until complications arise [4], [7], [8]. Conversely, causes of hemocholecysts include vascular disorders, malignancies, polyps, heterotopic gastrointestinal mucosa, and gallstones; among these causes, GBC is rare [1], [4]. The diagnosis of a hemocholecyst is also difficult due to its nonspecific clinical features. In our case, the hemocholecyst due to GBC caused anemia and precordial pain. Notably, precordial pain was the only symptom in our patient and this has not been reported previously as a symptom of hemocholecysts. Blood clot accumulation in the gallbladder may cause abdominal pain due to gallbladder distention. Although bile has fibrinolytic properties, small hemorrhages tend to form pure clots that do not contain gallstones and are likely to cause bile duct obstruction and cholecystitis [3]. As for hemorrhagic cholecystitis, its main etiologies are lithiasis and antiplatelet/anticoagulant medication; trauma, malignancy, renal failure, and cirrhosis have also been reported [9], [10]. Cholecystitis caused by clots with or without biliary calculi due to minor hemobilia should be distinguished from hemobilia resulting from mucosal erosion in the face of existing cholecystitis [3]. When hemorrhagic cholecystitis occurs, patients can present with abdominal pain, nausea, and vomiting similar to classic acute cholecystitis, as well as bile duct obstruction, hematemesis, or anemia, which can be life-threatening [10], [11], [12]. Although precordial pain is not reported as a symptom of hemorrhagic cholecystitis, a few cases of acute cholecystitis have been reported where patients presented with chest pain and ST segment elevation on ECG [13]. Abnormal ECG findings such as bradycardia and a 2nd-degree atrioventricular block may also be revealed within days of the onset of chest pain [13]. In our case, 9 h following the onset of precordial pain, abnormal ECG findings were still not noted. The problem with chest pain and abnormal ECG findings is that they raise suspicion of acute coronary syndrome and lead to additional close examinations, which may delay the diagnosis of cholecystitis [13]. Symptoms of acute coronary syndrome have been suggested to be due to the cardiobiliary vagal reflex [13], [14], [15]. In animal studies, gallbladder distention has been shown to cause reflex coronary vasoconstriction involving centrifugal sympathetic mechanisms associated with alpha-adrenergic receptors and afferent vagal pathways [15]. Furthermore, in human cases, intraoperative manipulation of the gallbladder (i.e., direct gallbladder stimulation) causes coronary artery spasms [14].

Except for gallbladder diseases, acute inflammatory and ulcerative conditions of the duodenum stimulate surrounding structures and may cause a reflex that limits or alters the blood supply to the coronary arteries through autonomic pathways. Since gastric distension, pericarditis, neoplastic invasion of the myocardium, acute cor pulmonale, and hypothermia can also cause chest pain with ST segment elevation, we must consider these diseases as differential diagnoses of precordial pain [16], [17].

CT is the most complete test for evaluating hemorrhagic cholecystitis [9]. Multiple fluid levels and increased bile density on CT may suggest blood in the gallbladder [10]. CT findings in patients with hemorrhagic cholecystitis include perivesicular inflammatory changes, dense gallbladder content due to hemobilia, hemoperitoneum, perihepatic hematoma, dense content in the bile duct or bowel, and active bleeding [9].

On T1WI magnetic resonance imaging (MRI), the hemorrhagic components are hyperintense; on T2WI MRI, the signal intensity may change based on the oxygen saturation of hemoglobin and time course [11]. A gallbladder mass can be identified on ultrasound sonography (US) or CT scan; however, it is difficult to confirm in the presence of a clot in the gallbladder lumen due to a hemocholecyst or hemorrhagic cholecystitis as the clot appears echogenic. Since not all cases will reveal a high-density mass and clinicians often do not assume an association between GBC and blood clots, GBC may be detected only during surgery [3].

Hemorrhagic cholecystitis requires early diagnosis and urgent cholecystectomy to prevent perforation [11], [12]. In cases such as intraluminal hemorrhage due to pseudoaneurysm formation and gallbladder artery rupture associated with gallbladder wall necrosis, urgent surgery is indicated [12]; microcoil embolization of the bleeding cystic artery followed by cholecystectomy is also effective when the patient is hemodynamically stable [8], [18]. Meanwhile, simple gallbladder hematomas due to trauma or bleeding diathesis can be treated conservatively [3].

In our case, the patient's severe anemia without dyspnea could be attributed to intermittent or occult gastrointestinal bleeding from the hemocholecyst [4]. Since the anemia was revealed, a CT scan was performed, leading to the diagnosis of a hemocholecyst. The only symptom that our patient experienced was precordial pain, which is thought to be due to cardiobiliary vagal reflex stimulation. This shows that precordial pain can occur without coronary artery disease. Although blood examination did not reveal an inflammatory reaction at the time the patient was admitted, increased pericholecystic attenuation on arterial-phase CT, which is an early manifestation of acute cholecystitis, suggested developing hemorrhagic cholecystitis [19]. As noted previously, we could not diagnose GBC preoperatively. While the preoperative imaging modality in our case was only CT, which did lead to the diagnosis of a hemocholecyst, contrast-enhanced MRI would have detected GBC and should have been performed. MRI could be useful in evaluating gallbladder soft tissue characteristics; contrast-enhanced MRI may be able to detect GBC in a case of a hemocholecyst containing T1 high-intensity clots by subtraction imaging [20].

## 4 Conclusion

A hemocholecyst should be considered as a differential diagnosis for chest pain, with GBC as an important cause of hemocholecysts. In line with this, there may be a role for abdominal imaging in the diagnosis of patients with nonspecific chest pain. Early diagnosis and urgent cholecystectomy should be performed to prevent gallbladder perforation in the case of a hemocholecyst.

## Provenance and peer review

Not commissioned, externally peer-reviewed.

## Consent

Written informed consent was obtained from the patient for publication of this case report and its accompanying images. A copy of the written consent is available for review by the Editor-in-Chief of this journal on request.

## Ethical approval

This case report was approved by our hospital's research ethics committee (Approval No. 2022-6).

## Sources of funding

None.

## Author contribution

GF performed the operation, manuscript writing and editing.

## Research registration number

This study was registered as a case report in the UMIN Clinical Trials Registry (https://www.umin.ac.jp/ctr/) with the unique identifying number UMIN000049040.

## Guarantor

Goshi Fujimoto.

## Patient perspective

The patient and her family understood the medical condition and the necessary treatment prior to the operation. Although the tumor depth was T2a, considering her age and general condition, the patient and her family decided to forgo additional gallbladder bed resection or S4/5 hepatic bisegmentectomy and lymphadenectomy.

## Availability of data and materials

The datasets supporting the conclusions of this article are included within the article.

## Declaration of competing interest

None.
